# Limited Evidence of Associations Between Executive Functioning and Alcohol Involvement In UK Adolescents

**DOI:** 10.1093/alcalc/agab020

**Published:** 2021-04-09

**Authors:** Sam Burton, Jo-Anne Puddephatt, Laura Baines, Florence Sheen, Jasmine G Warren, Andrew Jones

**Affiliations:** Department of Psychology, Institute of Population Health, Eleanor Rathbone Building, Bedford Street South, University of Liverpool, L69 7ZA, UK; Department of Psychology, Institute of Population Health, Eleanor Rathbone Building, Bedford Street South, University of Liverpool, L69 7ZA, UK; Department of Psychology, Institute of Population Health, Eleanor Rathbone Building, Bedford Street South, University of Liverpool, L69 7ZA, UK; Department of Psychology, Institute of Population Health, Eleanor Rathbone Building, Bedford Street South, University of Liverpool, L69 7ZA, UK; Department of Psychology, Institute of Population Health, Eleanor Rathbone Building, Bedford Street South, University of Liverpool, L69 7ZA, UK; Department of Psychology, Institute of Population Health, Eleanor Rathbone Building, Bedford Street South, University of Liverpool, L69 7ZA, UK

## Abstract

**Aims:**

Deficits in motor inhibitory control and working memory have been hypothesized to be both a cause and consequence of heavy alcohol use. Adolescence is a critical developmental stage for inhibitory control and working memory, and it is also a stage when individuals are most likely to initiate alcohol use. This study aimed to examine whether inhibitory control and working memory would predict alcohol use and involvement in a group of UK adolescents.

**Methods:**

We recruited 220 (*N* = 178, female) adolescents, aged between 16 and 18, from eight higher education settings in the Merseyside region of the UK. Alcohol use was examined using the Timeline Follow-Back and involvement (and related problems) using the Adolescent Alcohol Involvement Scale. A reward-based inhibitory control task (Go/No-Go) was used to examine the inhibition and reward sensitivity, and a self-ordered pointing task was used to measure working memory.

**Results:**

Multiple regression demonstrated that neither inhibitory control (*b* = 0.02 (95% confidence interval (CI): −0.21, 0.24)) nor working memory (*b* = −0.12 (95% CI: −0.30, 0.07)) were significant predictors of alcohol use (units consumed). Inhibitory control (*b* = 0.61 (95% CI: 0.12, 1.09), specifically, in the no reward condition and school deprivation (*b* = 0.67 (95% CI: 0.06, 1.28) significantly predicted alcohol-related problems.

**Conclusions:**

Our findings demonstrated limited evidence that deficits in specific mechanisms of executive functioning (i.e. motor inhibition and working memory) were associated with alcohol-related problems in UK adolescents. This study adds to an increasing body of literature suggesting weak or non-existent links between inhibitory control, working memory and alcohol use.

## INTRODUCTION

In the UK, initial experimentation with alcohol typically begins during early adolescence (Fernie *et al.*, 2013). Eight percent of 11-year-olds report consuming alcohol, which rises to 69% by age 15 (Hawkins, 2012). Alcohol consumption during adolescence is associated with a range of negative health outcomes, including neurocognitive deficits (Zeigler *et al.*, 2005), short-term physical harm and risky behaviours (Boden *et al*.*,* 2011). Furthemore, earlier onset of alcohol use is associated with increased risk of developing a substance use disorder in later life (Hingson and Zha, 2009). Encouragingly, recent work suggests that alcohol consumption is on the decline in youth drinkers in the UK (Oldham *et al.*, 2019), with similar findings from Europe and North America (Norstrom and Svensson, 2014; Raninen *et al.,* 2014; Looze *et al.*, 2015); however, the prevalence of adolescent drinking is still a concern, given the associations with a range of negative health outcomes (Zeigler *et al.*, 2005).

Adolescence is a key developmental stage for (executive) cognitive functions and impulsive behaviour. Broadly speaking, impulsivity can be viewed as the opposite of a general cognitive ability, with the two constructs overlapping both theoretically and in measurement instruments (Bickel *et al.*, 2012). Both constructs have been previously implicated as both the cause and consequence of excessive alcohol consumption (Coskunpinar *et al.*, 2013). Key components of both constructs are inhibitory control and working memory (Miyake *et al.*, 2000; Bickel *et al.*, 2012). Inhibitory control is the ability to control or adjust one’s behaviour in response to internal or external factors (Logan *et al.*, 1984; Diamond, 2013), and it is multifaceted, encompassing a variety of conscious and subconscious behaviours, such as memory, attention and motor movements (Diamond, 2013). Computerized tasks have been developed to objectively assess motor inhibitory control, such as stop signal tasks (SSTs) and Go/No-Go, in which a dominant motor response is established and participants are required to inhibit this response on a minority of trials (Logan and Cowan, 1984; Diamond, 2013). Luna *et al.* (2004) suggest that basic-level response inhibition, voluntary initiation and suppression of behaviours are present in early childhood and they develop further during adolescence.

Working memory is a cognitive system which enables the provisional storage of information, no longer perceptually present, working with said information for complex cognitive abilities even in the presence of distractors (Baddeley, 1992; Engle, 2002). Working memory can be assessed using tasks such as the self-ordered pointing task (SOPT) (Petrides and Milner, 1982). Both inhibitory control and working memory are thought to develop during adolescence (Luna *et al.*, 2004; Blakemore and Choudhury, 2006; Casey *et al.*, 2008). Miyake and Friedman (2012) review of executive functions states both inhibitory control and working memory demonstrate unity and diversity (they are correlated, yet separable; see also Diamond, 2013). Executive function deficits have been previously associated with risk-related behaviour, with arguments made for slower maturation of the prefrontal cortex in adolescence along with developing cognitive control leading to risky behaviours (Steinberg, 2007; Casey *et al.*, 2008).

Adolescence is also a period of increased reward sensitivity, which is associated with alcohol use across adolescent populations (Knyazev, 2004; Pardo *et al.*, 2007; Lopez-Vergara *et al.*, 2012). Heightened reward sensitivity has been operationalized as increased impulsive decision-making and decreased inhibitory control to rewards (Peeters *et al.*, 2017). Findings suggest that reward sensitivity can promote adolescent alcohol use, with reactivity to rewarding cues able to predict current (van Hemel-Ruiter *et al.*, 2013) and future alcohol use (van Hemel-Ruiter *et al.*, 2015). Extrinsic motivation (through explicit rewards) can facilitate inhibitory control in both healthy and heavy drinking samples (Chung *et al.*, 2011; Schevernels *et al.*, 2014; Wilbertz *et al.*, 2014; Schevernels *et al.*, 2015) and adolescent samples (Kohls *et al*., 2009b; Winter and Sheridan, 2014; Demurie *et al.*, 2016). From a neuroeconomics perspective, the use of extrinsic reward stimuli may increase the attributed value of inhibiting behaviour (Guttman *et al.*, 2018) and may provide a more comprehensive representation of the psychological mechanism of inhibitory control (Poulton *et al.*, 2016).

Within individuals who drink alcohol, individual differences in impulsivity/executive functioning have been shown to be associated with the quantity and frequency of consumption and related problems, escalation of use (Fernie *et al.*, 2013; Bø *et al.*, 2017) and transition to heavy drinking within adolescence (Wetherill *et al.*, 2013). Elevated levels of impulsivity and motor disinhibition can pre-date alcohol involvement (and related problems) acting as a potential risk factor for heavy drinking and dependence following experimentation (Dawe *et al.*, 2004; Ersche *et al.*, 2012; Whelan *et al.*, 2014) in adult samples. Some researches suggest the relationship between alcohol use and impaired executive functions to be weak or non-existent (MacKillop *et al.*, 2007; Balodis *et al.*, 2009; Peeters *et al.*, 2014; Caswell *et al.*, 2016). It is possible that individual and methodological differences across studies account for discrepant findings. For example, inconsistencies may be the result of the measure used (Smith *et al.*, 2014; Fernandez-Artamendi *et al.*, 2018). Deficits in inhibitory control are suggested to be dose-dependent, with deficits appearing to be smaller in heavy non-dependent drinkers compared to dependent drinkers (Smith *et al.*, 2014), particularly, in females (Nederkoorn *et al.*, 2009; Smith and Mattick, 2013; Smith *et al.*, 2015).

Inhibitory control is thought to fluctuate in response to environmental, psychological and physiological cues acting as transient state triggers (De Wit, 2009; Jones *et al.*, 2013; Inzlicht and Berkman, 2015). Heavy drinking episodes are often triggered by alcohol-related cues (e.g. sight of alcohol) causing transient impairments of motor inhibitory control (Gauggel *et al.*, 2010; Ryan *et al.*, 2010), increasing craving and alcohol-seeking behaviours (Christiansen *et al.*, 2017). Evidence points to motor inhibitory control mediating the relationship between alcohol-cue exposure and subsequent alcohol consumption (Field and Jones, 2017). To our knowledge, no study has examined the effect of alcohol-specific impairments in inhibitory control or working memory in adolescence. It is possible that that any association between executive functions and alcohol use (or involvement) is better explained by exposure to alcohol-related cues (or alcohol context) than by non-alcohol-related cues due to their potentially compromising effects. Indeed, ‘hot’ executive functions (those linked to emotional responding and reward sensitivity) are better linked to risky behaviours in adolescence (Prencipe *et al.*, 2011). However, it is also possible that due to a shorter drinking history, adolescents may be less sensitive to exposure to alcohol-related cues.

Therefore, the aim of the present study was to examine the role of motor inhibitory control, reward sensitivity and working memory on alcohol-related problems in adolescents in the Merseyside area of the UK through executive function measures containing alcohol-related cues. We hypothesized that (a) individual differences in motor inhibitory control will predict alcohol-related problems, with reward-specific inhibitory control predicting unique variance, (b) individual differences in working memory will predict alcohol-related problems and (c) individuals will have lower commission errors on the Go/No-Go task in the reward condition compared to the no reward condition. Hypotheses were pre-registered on Open Science Framework (https://osf.io/yd9ua).

## MATERIALS AND METHODS

### Participants

Two hundred and twenty participants (*N* = 220/18.75% male) were recruited from psychology courses across eight further education centres in the Merseyside area of the UK. Participants were eligible to take part if they were aged between 16 and 18 years of age (mean age = 16.73 years, standard deviation (SD) = 0.68), had no previous or current diagnosis of substance use, attention deficit hyperactivity disorder (ADHD) and/or psychiatric or neurological disorder (16 participants were removed based on this criteria). Participants were asked if they had a current or previous diagnosis of the aforementioned, by indicating yes or no on a check box. Inclusion/exclusion criteria were assessed via self-report. All participants provided informed consent, and both parents and further education centres received an information pack with details of the study prior to commencement. At the time of data collection in the Merseyside region, 20% of the population were considered to be among the most deprived in the UK (Taib *et al.*, 2018), with 31.8% of children living in poverty in the region (Stone and Hirsch, 2019), 27.6% of adults in the region drinking over the recommended government guidelines and 20.2% binge drinking, both above the average for UK (Public Health England). The University of Liverpool Research Ethics Committee approved the study.

Our sample size was constrained to the availability and willingness of higher education institutions to be involved in the research. However, our informal power calculation suggested 187 participants were needed to detect an *R*^2^ increase of 0.05 (explained by Inhibitory Control and Working Memory as tested predictors) and four covariates (age, sex, scores on the Family Affluence Scale (FAS) and school deprivation) at 80% power. We decided on an *R*^2^ increase of 0.05 as Henges and Marczinski (2012) reported a correlation of *r* = 0.22 (*R*^2^ = 0.048) between inhibition failures and the total number of drinks consumed by 108 young social drinkers.

### Self-report measures

#### Demographics and socio-economic status

Participants reported their gender and age before completing the six-item FAS (Currie *et al.,* 1997). Questions required participants to report on ownership of family car(s), whether they have their own bedroom, number of computers in the home, number of bathrooms, etc. FAS is a well-validated measure of socio-economic status (SES) in ages as young as 11 years old, and it has been shown to correlate well with other measures of SES, such as disposable income (Torsheim *et al.*, 2004; Hobza *et al.*, 2017). Scores ranged from 0 to 6 (higher score indicative of higher SES), with a mean score of 3.52 (SD = 1.40).

#### Alcohol use

Participants completed a 2-week retrospective diary of all alcoholic beverages they consumed, Timeline Follow-Back (TLFB) (Sobell and Sobell, 1992) to assess the frequency and quantity of alcohol consumption. Participants were asked to record the number of units they consumed on a daily basis for the previous 14 days. A guide of units was provided for standard measurements of a variety of drinks, e.g. a small glass of wine or bottle of beer. Total units consumed during the previous 14 days and binge drinking frequency were the outcome measures.

#### Alcohol Involvement Scale

Participants completed the Adolescent Alcohol Involvement Scale (AAIS) (Mayer and Filstead, 1979), a 14-item self-report questionnaire measuring alcohol abuse and alcohol-related problems. Questions are rated on a seven-point Likert-type scale, with a total possible score of 79. Options at the lower end are anchored at 0, e.g. Question 2, ‘When did you last drink alcohol?’, 0 = never used alcohol and 7 = today. The 14 items are deemed to share sufficient common variance to create a composite alcohol use score (McKay and Dempster, 2016).

#### Index of Multiple Deprivation

For each school, the level of deprivation was coded according to the Index of Multiple Deprivation (IMD) (Noble *et al.*, 2006). IMD classifies deprivation based on the proportion of deprived individuals in an area (Cemlyn *et al.*, 2002; Noble *et al.*, 2006). School deprivation scores ranged from 1 to 8, with 1 being high deprivation and 8 being low deprivation.

### Behavioural measures

#### Go/No-Go task

A hypothetical reward Go/No-Go task was administered (Demurie *et al.*, 2016), consisting of 224 trials, of which 75% (*N* = 168) were Go trials and 25% were (*N* = 56) No-Go trials, with half of all trials being rewarded. The fixation cross presented at the start of a trial for 500 ms, and the colour of the cross denoted if experiment ‘points’ (the reward) could be won for a correct response or not (yellow = reward, blue = no reward). Point-based rewards have been used in previous inhibitory control studies, and participants respond with motivation to obtain these points, as they would to a reward with actual monetary value (e.g. Geier *et al*., 2012; Marx *et al*., 2013; Miyasaka *et al*., 2019). Go and No-Go stimuli were presented on screen for up to 2000 ms. On Go trials participants were shown images of soft drinks whereby they had to press the space bar as quickly as possible, while No-Go cues were images of alcohol drinks where they had to refrain from pressing the space bar. Between each trial was an inter-trial interval of 1000 ms. Average Go reaction time (RT) and commission errors were calculated for both reward and no reward conditions. Before completing the task, participants were given a brief on the instructions, with 20 practice trials which could be repeated if necessary.

#### Self-ordered pointing task

A modified SOPT was used to assess working memory (Petrides and Milner, 1982), which has been used in adolescences in relation to substance use previously (Thush *et al.*, 2008; Bourque *et al.*, 2016; Carbia *et al.*, 2017). We used alcohol—rather than neutral-related—images to invoke cue-exposure and ensure consistency with the Go/No-Go task. Participants were shown a set of alcohol-related images (e.g. glass of beer), displayed in an array (grid format), and were asked to select one using their mouse. Following the selection of an image, a new page is displayed with the previous images, and all images were automatically re-arranged into different positions. Participants were asked to select an image, while avoiding clicking the same image in a block and avoid clicking the same position in the array. There were three blocks of 6 (2 × 3 array), 8 (2 × 4 array), 10 (2 × 5 array) and 12 (3 × 4 array) image arrays. The number of trials for each block was in accordance with the number of images in the array. Between each trial was an inter-trial break of 1000 ms. At the end of all blocks, participants were told their total number of errors, as a measure of working memory. The SOPT has been shown to demonstrate good psychometric properties and is related to other measures of working memory (Cragg and Nation, 2007; Ross *et al.*, 2007).

### Procedure

Schools were visited during the months of March–December 2019. Multiple visits to each school to assess longitudinal associations were planned, however, we were unable to do this due to the COVID-19 pandemic. Our procedure for testing was identical across all schools. Before the visit, schools were sent information sheets and we discussed the study with the lead author. Parents and guardians were informed about the study at least 1 week before the scheduled site visits. Consent was obtained on site from the students in line with British Psychological Society guidelines, as all participants were aged 16+. Participants were either tested at their school or at the University of Liverpool, with group sizes ranging from 10 to 20 participants and multiple researchers were present. All participants sat at individual computers or laptops to complete the experiment. Participants completed the battery of questionnaires, followed by the Go/No-Go task and SOPT. Upon completion of the study, participants and teachers were debriefed as to the purpose of the study.

### Data reduction and analysis

Data were cleaned and analysed in R, using the ‘dplyr’ and ‘lme4’packages (r-script can be found on OSF). Average Go RT was calculated for both reward and no reward conditions. Outliers were identified using box plots and were removed from individual analyses. RT data from one individual was removed due to non-responding on Go-Trials. Ten participants (4.54%) were removed for outlying commission errors. Commission errors were calculated for reward and non-reward trials along with an overall number of commission errors. Total errors were recorded on for SOPT as the measure of working memory.

We examined whether multi-level modelling was appropriate for data analysis due to the use of nested data (individuals > schools). These models were not a better fit of the data—however, this is consistent with Fernie *et al.*’s (2013) data, which indicated any clustering effect of school effects was nominal, as such we used standard linear regression analyses.

## RESULTS

### Participant demographics

Demographic information for the complete sample stratified by school is reported in Table 1. Of the 220 participants, 57.73% had consumed alcohol in the previous 2 weeks to the testing sessions. Fifteen (6.82%) of the participants were classified as heavy drinkers, in accordance with UK guidelines, having drunk 28 or more units over a 2-week period. One individual reported drinking an implausible amount (265 units) as such we rescaled this to the next largest value +1. Average consumption was 14.01 units (SD = 13.32: range 1–73).

**Table 1:** School characteristics, means and SDs

School code	1 *N* = 20	2 *N* = 95	3 *N* = 20	4 *N* = 23	5 *N* = 11	6 *N* = 10	7 *N* = 26	8 *N* = 15	Total *N* = 220
School deprivation (IMD)	3	1	3	8	4	4	1	8	
Male, %	15.00	0	100.00	47.82	27.27	100	0	0	18.75
Age	16.80 (0.52)	16.55 (0.58)	16.4 (0.50)	16.57 (0.59)	17.71 (0.30)	16.90 (0.32)	17 (0.49)	16.8 (0.41)	16.73 (0.68)
TLFB total	4.75 (12.00)	6.25 (8.61)	7.46 (12.60)	11.23 (15.74)	21.36 (22.93)	16.5 (17.37)	0.5 (2.16)	5.87 (9.05)	8.95 (21.39)
AAIS	29.65 (14.62)	28.57 (11.15)	31.45 (9.48)	34.74 (7.31)	33.64 (13.44)	31 (8.98)	25.04 (11.65)	29.87 (6.74)	29.76 (11.12)
FAS	3.25 (1.59)	3.82 (1.38)	3.2 (1.28)	3.13 (1.33)	3.27 (1.85)	3.8 (1.14)	2.88 (1.42)	3.67 (0.82)	3.49 (1.40)

#### The effect of reward on inhibitory control

 analyse the effect of reward on inhibitory control, we conducted a paired samples *t*-test on commission errors in reward and no reward conditions. There was a significant difference in the commission errors between the reward (*M* = 6.48, SD = 4.43) and no reward (*M* = 5.18, SD = 3.82) conditions (*t*(209) = 4.84, *P* < 0.001, *d* = 0.31), and this result remained significant with commission error outliers in the sample (*t*(219) = 4.97, *P* < 0.001). In exploratory analysis, we analysed the effect of reward on Go RTs, using a paired *t*-test. There was a significant difference between go RTs in reward (*M* = 435.29, SD = 49.27) and no reward (*M* = 443.53, SD = 63.85) conditions (*t*(209) = 3.16, *P* < 0.01, *d* = 0.15), this result was non-significant with commission error outliers in the sample (*t*(219) = 1.03, *P* = 0.306).

#### Predicting individual alcohol use (TLFB)

A multi-level model was not a significantly better fit for the data than the single-level model (χ^2^(1) = 3.34, *P* > 0.05), as such ordinary least squares multiple regression was used to analyse the data, see Table 2. Model A included commission errors and SOPT errors as predictors. Model A did not explain a significant amount of variance in the data, *F*(2,207) = 0.365, *P =* 0.695, BF_01_ = 22.75, adjusted *R*^2^ < 0.01, with Bayes factors ranging from 0.15 to 0.19. In Model B, commission errors was split into reward and no reward, with SOPT errors as predictor variables. Model B did not significantly account for the variance in TLFB data, *F*(3,206) = 0.700, *P =* 0.553, BF_01_ = 49.59, adjusted *R*^2^ < 0.01, Bayes factors ranging from 0.15 to 0.27. In Model C, we included the variables from Model A and included, age, gender, FAS and school deprivation as covariates. In Model C, commissions errors were not split into reward or no reward commissions due to no significant association in Model B. Model C explained 12.46% of variance in the data, *F*(6,203) = 5.960, *P* < 0.001, BF_01_ = 0.12, adjusted *R*^2^ = 0.125. Gender (β = −9.31 (95% confidence interval (CI): −13.50 to −5.13), *P <* 0*.*001) and age (β = 3.19 (95% CI: 0.83–5.54), *P <* 0*.*01) were significant predictors, suggesting that as age increased so did the alcohol consumption. Males consumed significantly more units of alcohol (*M* = 15.30, SD = 18.4) than females (*M* = 5.34, SD = 9.38, *t*(49) = 3.49, *d* = 0.88). There was limited evidence of multicollinearity across the three models (VIFS < 1.76). For sensitivity analysis, models were ran without outliers removed, and the results did not differ for any of the models reported above. A logistic regression in which individuals who reported drinking versus not drinking as the outcome did not substantially change the pattern of results, nor a model in which only alcohol consumers was included in the analysis.

**Table 2:** Unstandardized beta values and 95% CIs indices for multiple regression models with TLFB consumption as the dependent variable

					Predictor variable							
	*F*	Adjusted *R^2^*	*P* value	BF_01_	Reward errors	No reward errors	Total errors	SOPT errors	Gender	Age	FAS	School deprivation
Model A	0.364	<0.01	0.695	22.75			0.05 (−0.19, 0.29)	−0.08 (−0.28, −0.11)				
Model B	0.700	<0.01	0.553	49.59	−0.19 (−0.66, 0.28)	0.34 (−0.21, 0.88)		−0.09 (−0.29, 0.10)				
Model C	5.960	0.125	<0.001	0.12			0.02 (−0.21, 0.24)	−0.12 (−0.30, 0.07)	**−9.31 (−13.50, −5.13)**	**3.19 (0.83, 5.54)**	0.20 (−0.99, 1.38)	0.33 (−0.32, 0.98)

Note: errors = commission errors; predictors in bold are significant.

#### Adolescent Alcohol Involvement Scale

A multi-level model was not a significantly better fit for the data than the single-level model (χ^2^(*df* = 1) = 2.00, *P >* 0.05), as such a multiple regression was used to analyse the data, see Table 3. In Model A, we included SOPT errors and commission errors as predictors. The multiple regression model was not significant, *F*(2,207) = 1.617, *P* = 0.201, BF_01_ = 6.66, adjusted *R*^2^ = 0.005, accounting for 0.5% of variance in the data. Model B was run with commission errors split into reward and no reward conditions and SOPT errors. The model was not significant, *F*(3,206) = 1.95, *P* = 0.123, BF_01_ = 7.63, adjusted *R*^2^ = 0.013, accounting for 1.3% of variance in the data. However, no reward errors were significantly associated with the AAIS score (β = 0.53 (95% CI: 0.04–1.02), *P =* 0*.*033), and commission errors were thus split into reward and no reward commissions in Model C. Results for Model C (including covariates) suggest that no reward commission errors and school deprivation predict 5.05% of the variance (*F*(7,202) = 2.59, *P =* 0*.*014, BF_01_ = 3.42, adjusted *R*^2^ = 0.050). As no reward commission errors increased, AAIS score increased (β = 0.61 (95% CI: 0.12–1.09), *P =* 0*.*014). School deprivation had a significant relationship with AAIS score (β = 0.67 (95% CI: 0.06–1.28), *P* = 0.031); as deprivation decreased, AAIS score increased. There was limited evidence of multi-collinearity across the models (VIFs < 1.78) For sensitivity analysis, models were ran without outliers removed, and the results did not differ for any of the models reported above.

**Table 3:** Unstandardized beta values and 95% CIs for multiple regression models with AAIS as the dependent variable

					Predictor variable							
	*F*	Adjusted *R^2^*	*P* value	BF_01_	Reward errors (95% CI)	No reward errors (95% CI)	Total errors (95% CI)	SOPT errors (95% CI)	Gender (95% CI)	Age (95% CI)	FAS (95% CI)	School deprivation (95% CI)
Model A	1.617	0.005	0.201	6.66			0.17 (−0.04, 0.39)	−0.11 (−0.28, 0.07)				
Model B	1.95	0.013	0.123	7.64	−0.11 (−0.53, 0.30)	0.**53 (0.04, 1.02)**		−0.11 (−0.29, 0.06)				
Model C	2.59	0.050	0.014	3.42	−0.15 (−0.57, 0.26)	**0.61 (0.12, −1.09)**		−0.12 (−0.29, 0.06)	−2.15 (−6.07, 1.78)	1.94 (−0.27, 4.15)	−0.01 (−1.12, 1.09)	0.**67 (0.06, 1.28)**

Note: errors = commission errors; predictors in bold are significant.

## DISCUSSION

In the present study, we examined if measures of executive function—motor inhibitory control and working memory—were associated with alcohol-related problems or consumption in a sample of adolescents. We also examined whether reward sensitivity interacted with motor inhibitory control to predict unique variance in alcohol-related problems. We found a significant association between no reward commission errors and alcohol-related problems, yet no other measures of executive functioning were significant for alcohol-related problems or consumption. In the presence of a reward, motor participants’ inhibitory control was significantly poorer.

Contrary to our hypotheses, motor inhibitory control (as measured by commission errors on a Go/No-Go task) was not associated with alcohol consumption, with Bayesian analysis-suggesting findings as evidence for the null hypothesis. Motor inhibitory control performance, in no reward conditions, was associated with alcohol-related problems. Specifically, as motor inhibitory control became poorer, alcohol-related problems increased. These findings provide limited support for theoretical models or empirical data which suggest motor inhibitory control and working memory are associated with alcohol involvement (Field and Jones, 2017; Carbia *et al.*, 2018; Mahedy *et al.*, 2018). The lack of a consistent association across different studies may be due to the precision of the measure of inhibitory control administered and the samples used (e.g. lighter vs. heavier drinkers).

The presence of reward increased commission errors, an effect which in isolation is unexpected (Chung *et al.*, 2011; Schevernels *et al.*, 2014; Wilbertz *et al.*, 2014). However, there is evidence that reward can impair inhibition (Kohls *et al*., 2009a; Padmanabhan *et al.*, 2011; Demurie *et al.*, 2016; Miyasaka and Nomura, 2019), and it is possible that prompting reward on Go trials led to faster RTs (which we observed in comparison to non-rewarded trials), which in turn, increased inhibition errors due to a speed–accuracy trade-off (Leotti and Wager, 2010). Alternatively, Pessoa (2009) suggests that a deleterious effect of reward on inhibitory control is the result of a (finite) resource allocation to maximize the chance of reward, causing other cognitive systems to suffer. The effect of reward should be examined with different reward types (e.g. hypothetical or actual, financial or non-financial), as evidence suggests reward salience changes as age develops (Miyasaka and Nomura, 2019). As such, the current reward may not have been sufficiently salient to the participants.

Interestingly SES, reported through the FAS, did not explain a significant proportion of the variance in drinking behaviour among adolescence. This is in line with previous work, which shows no clear pattern between the drinking behaviour and SES in adolescence (Hanson and Chen, 2007). School-level deprivation scores did explain a significant proportion of drinking behaviour, but there was not a significant difference between school deprivation. This may be explained by the difference in the number of participants recruited from each school deprivation group, which was assigned based on the school postcode.

Findings from the current study should be assessed in light of limitations. Our sampling was limited to one geographical area in the UK, characterized by greater-than-average deprivation. Future studies should attempt to recruit from multiple geographical locations to increase the representativeness of these findings. Second, a 2-week TLFB may not have been sufficiently long enough to capture alcohol consumption in adolescents, as access to alcohol in these samples may be varied (Jones-Webb *et al.*, 1997). Future research should attempt to replicate these findings using measures of alcohol use over longer time periods (Buu *et al*., 2014). In relation to this, self-reported consumption may be prone to memory biases and under-reporting (Livingston and Callinan, 2015). Third, due to testing constraints, we were unable to assess other factors which might be related to both executive functioning and alcohol use, such as impulsive personality traits and mental health. Fourth, we used an unbalanced Go/No-Go design to assess the inhibitory control. Future research should use a counter-balanced Go/No-Go design (in which the contingency for responding/inhibiting to neutral cues is reversed) to disentangle any attentional bias towards alcohol-related cues. Similarly, we combined reward sensitivity and inhibitory control within the Go/No-Go task, which is both a strength and limitation, as it provides a more realistic outcome given the interdependency of these processes but limits direct conclusions for either in isolation. Finally, we originally aimed to conduct follow-up assessments for each participant, however, these were unable to take place due to the COVID-19 pandemic and so we are limited to cross-sectional associations. Examination of prospective associations throughout adolescence may demonstrate different results (e.g. Fernie *et al.*, 2013).

Findings from the current study have implications for both alcohol research in adolescents and examination of executive functioning in this population. The majority of models and empirical studies hypothesize an overly simplistic association between the two variables, with varying degrees of support (see Wiers *et al.*, 2010; Fernie *et al.*, 2013). However, inhibitory control is sensitive to a number of inputs, including reward and motivation, and in order to make clearer predictions about behaviour, the interactions between inhibitory control and external/internal inputs should be modelled. This is the first study, to our knowledge, to examine more complex relationships between inhibitory control and alcohol consumption in such a manner with this population.

To conclude, this study found limited evidence of associations between measures of executive functioning (motor inhibition and working memory) and alcohol use/involvement in adolescents. This adds to a growing number of studies which suggest that the link between inhibitory control (and working memory) and alcohol use is weaker than first thought. To more accurately examine the role of executive functioning on alcohol use, future studies should use multiple measures of constructs of executive functioning, allowing for multiple of individual associations and a combined composite measure.
